# A Case of an Epithelioid Hemangioendothelioma Arising from the Innominate Vein Mimicking Cervical Metastatic Lymphadenopathy

**DOI:** 10.1155/2016/4238575

**Published:** 2016-12-12

**Authors:** Jason B. Brill, Isaac E. Schwartz, Lindsey M. Prescher, Theodore C. Pratt

**Affiliations:** ^1^Department of General Surgery, Naval Medical Center San Diego, San Diego, CA, USA; ^2^Department of Otolaryngology, Naval Medical Center San Diego, San Diego, CA, USA; ^3^Department of Cardiothoracic Surgery, Naval Medical Center San Diego, San Diego, CA, USA

## Abstract

*Background.* Epithelioid hemangioendothelioma (EHE) is a rare tumor usually presenting in soft tissue. EHE is a vascular malignancy of intermediate clinical behavior, with a histologic appearance of endothelial cells growing in nests or cords. Although EHE often originates from a vessel, it is relatively rare for a primary vascular EHE to originate from a large vein or artery. Occurrence in the mediastinum is exceptionally rare. There are no known associations with other malignancies.* Case Presentation.* We present a case of mediastinal invasive EHE in a 39-year-old female with concurrent papillary thyroid cancer. She initially presented with a thyroid mass found by her primary care provider, with preoperative imaging concerning for extension into the superior mediastinum. Operative exploration revealed a mediastinal mass distinct from her thyroid carcinoma with invasion into the great vessels, requiring off-pump interposition graft bypass for en bloc resection. Final pathology confirmed pT3N1b multifocal papillary thyroid carcinoma with a separate grade 1 pT1b EHE. Review of the literature describes the demographics, updated pathologic outcomes, histologic findings, and reported incidence of EHE.* Conclusions.* This is the first reported case of thyroid malignancy with separate and concurrent EHE. Surgeons should remain aware of this entity given its variable behavior. Although initially described as an indolent neoplasm, tumors with poor prognostic factors have been shown to be locally aggressive.

## 1. Background

Epithelioid hemangioendothelioma (EHE) is a rare, malignant, and oftentimes aggressive vascular tumor with marked clinical variability. It is most often found in the extremities, but approximately 8% of the time it can be found in the mediastinum [1]. Although EHE usually originates from a vessel, it is relatively rare for a primary vascular EHE to originate from a large vein or artery [2]. We describe a case of EHE encasing the right innominate vein, superior vena cava (SVC), and right internal jugular (IJ) vein, discovered intraoperatively during thyroid resection for papillary carcinoma.

## 2. Case Presentation

**Figure 1:**
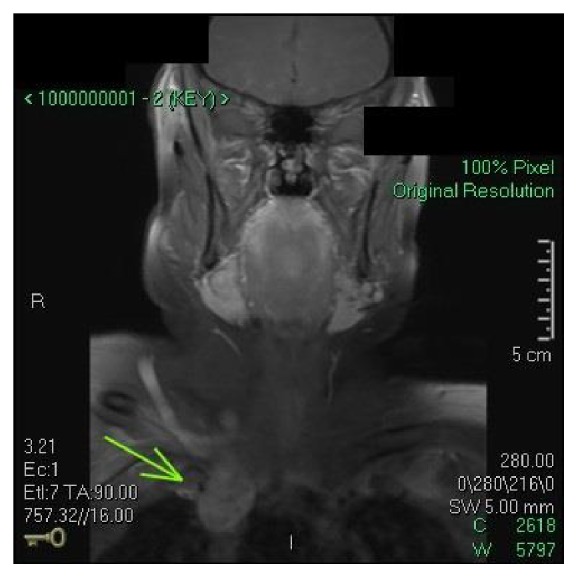
Coronal section from the preoperative magnetic resonance images showing a superior vena cava mass, presumed to be lymph nodes abutting the vein.

**Figure 2:**
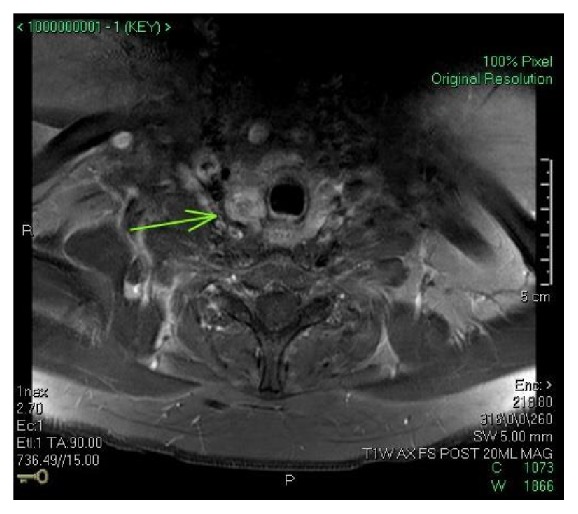
Axial section from the preoperative magnetic resonance images showing the same mass.

A 39-year-old woman was referred to the Otolaryngology Clinic at our facility for evaluation of a palpable thyroid mass, found during routine physical exam by her primary care physician. Initial work-up included thyroid functioning tests, which revealed normal levels of thyroxin and thyroid stimulating hormone. An ultrasound demonstrated a 2 cm heterogeneous-appearing right-sided nodule with internal microcalcifications, along with multiple small left sided nodules. Cervical lymph nodes were prominent but without concerning features on ultrasound. Fine needle aspiration confirmed papillary thyroid carcinoma (PTC) with features suggestive of the tall cell variant, a more aggressive variant of PTC [3]. Preoperative multiplanar magnetic resonance imaging (MRI) was obtained, revealing pathologic-appearing lymph nodes present in neck levels II, III, IV, V, and VII. A presumed cluster of nodes abutted the SVC (Figures 1 and 2). Positron emission tomography with computed tomography (PET/CT) showed locoregional metabolic activity with no evidence of distant metastasis. Given these findings, the patient underwent total thyroidectomy, bilateral central and lateral modified radical neck dissections (levels II–V), and superior mediastinal dissection. The right anterior mediastinal mass seen on MRI and presumed preoperatively to be a large metastatic lymph node was found to be densely adherent to the right internal jugular vein. Proximal to the mass, the vein was markedly dilated compared to the contralateral side.

**Figure 3:**
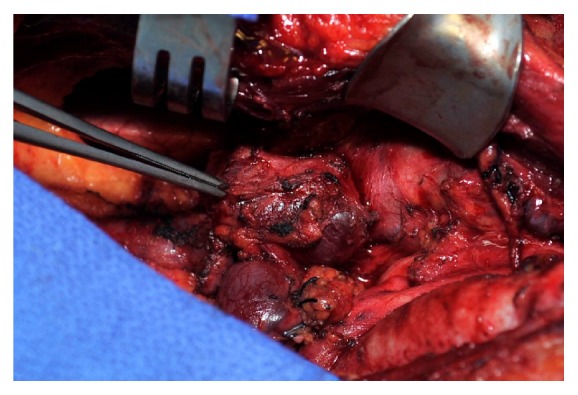
Intraoperative photograph showing an invasive mass obstructing the superior vena cava at the confluence of the right internal jugular and right innominate vein. Patient's head is to the right, and patient's right is at the top of the image.

A full sternotomy was perfor med, and the mass was dissected inferiorly as it appeared to be invading the confluence of the right IJ and right innominate veins (Figure 3). Vascular control was obtained, and, given the amount of encasement and invasion into the surrounding structures, en bloc resection was completed. The patient was heparinized and a 14 mm Dacron graft was selected for size and fashioned to bypass the right innominate vein to the right atrial appendage. The patient was discharged home on postoperative day seven following an uncomplicated hospital course.

Final pathology revealed multifocal papillary thyroid carcinoma with 4 of 164 lymph nodes positive for metastasis (pT3N1b). A 3.2 × 3.5 × 2.2 cm grade 1 EHE with lymphovascular invasion, 3 mitoses per 50 high powered fields (HPF), and scant necrosis (pT1bNx) was also reported. The tumor cells were diffusely positive for CD31, CD34, and vimentin, arranged in cords and clusters, and invasive into the lumen of the large vessels taken as part of the specimen. Given the strong CD31 immunohistochemical response and typical cellular architecture, it was concluded that this specimen represented EHE rather than thyroid carcinoma, which does not exhibit such endothelial markers [4]. Postoperative PET/CT at 6 weeks displayed no abnormal hypermetabolic areas and a patent graft. The patient has no evidence of recurrence now at one year after operation.

## 3. Literature Review

Epithelioid hemangioendothelioma was first described in 1982 by Weiss and Enzinger [5]. Within the thorax, pleural EHE is reported more frequently than mediastinal disease, most of which involves at least the anterior mediastinum. Invasion into the IJ, innominate, and azygous veins, as well as the superior vena cava, has been described [6–9]. EHE of the thyroid itself has been reported [10–12], although no association between thyroid malignancy and EHE has been described thus far. No cases exist in the available literature to date of a concomitant thyroid carcinoma and EHE.

Macroscopic and histologic appearances can vary, although stains for endothelial markers will often confirm the diagnosis. Visually, EHE is described as red-white, when associated directly with a vessel, to gray-white when distinct from nearby vessels [13]. Histologic features include cords and/or nests of epithelioid endothelial cells within a myxohyaline or chondroid stroma. Classically, “blister cells” show evidence of intracytoplasmic lumina, thought to be an intracellular attempt to produce vascular-like structures. The presence of necrosis, atypia, and increased mitotic activity differentiates intermediate-grade from low-grade EHE [14]. Immunohistochemistry can be confirmatory. Factor VIII-related antigen and cytokeratin CD34 are the typical endothelial markers, with CD31 being the most sensitive and specific marker for EHE. Ulex europaeus lectin and vimentin can be positive, as well [15]. Fluorescent in situ hybridization (FISH) has further increased the ability to classify EHE. Anderson et al. reported a case series displaying CAMTA1-WWTR1 gene fusions, which allowed the authors to differentiate EHE from other vascular tumors, especially useful for biopsies with limited tissue yield [14].

EHE carries an intermediate prognosis, between that of benign angioma and malignant angiosarcoma. Suster et al. reported a series of 12 cases of anterior mediastinal EHE which displayed indolent behavior [16]. Other series have reported more wide-ranging results. In the Anderson et al. series above, 4-year survival was 75% for low-grade EHE but only 9% for grade 3 [14]. For mediastinal tumors specifically, poor preoperative prognostic factors include size >3 cm, malignant pleural effusion, and clinical symptoms related to compression or obstruction of vascular structures. Concerning features for recurrence were described by Deyrup et al. in a series of 49 patients. Those with greatest tumor diameter >3 cm and increased mitotic activity (>3 per 50 HPF) experienced 59% survival with 32% recurrence at 5 years versus no deaths and 15% recurrence when these traits were not seen [1]. Higher grade and mitotic rates also warn of more aggressive tumor behavior [5, 13].

## 4. Conclusions

EHEs represent a seldom-encountered type of malignant vascular tumor even more rarely occurring in the mediastinum. Although case reports of great vessel invasion have been described, the literature has not previously documented thyroid carcinoma with EHE. Cellular markers and FISH can establish the diagnosis on fine needle aspiration. Though initially described as an indolent neoplasm, tumors with poor prognostic factors have been shown to be locally aggressive. Poor prognostic factors include size, associated symptoms, higher grade, and increased mitotic activity. While EHE within the thyroid gland has been described, this is the first reported case of concurrent thyroid malignancy with a separate mediastinal EHE.
